# Validity and reliability of the Spanish version of the 10-item CD-RISC in patients with fibromyalgia

**DOI:** 10.1186/1477-7525-12-14

**Published:** 2014-02-01

**Authors:** Blanca Notario-Pacheco, Vicente Martínez-Vizcaíno, Eva Trillo-Calvo, Mari Cruz Pérez-Yus, Dolores Serrano-Parra, Javier García-Campayo

**Affiliations:** 1 Social and Health Care Research Center, University of Castilla-La Mancha, Edificio Melchor Cano Santa Teresa Jornet Street, 16071 Cuenca, Spain; 2 Miguel Servet University Hospital, Zaragoza, Spain; 3 REDIAPP: Network on Preventive Activities and Health Promotion, Barcelona RD06/0018/0017, Spain, Aragon’s Institute of Health Sciences, Zaragoza, Spain; 4 Faculty of Nursing, University of Castilla-La Mancha, Cuenca, Spain; 5 Department of Psychiatry, Miguel Servet Hospital. REDIAPP: Network on Preventive Activities and Health Promotion, Barcelona RD06/0018/0017, Aragon’s Institute of Health Sciences, Cuenca, Spain

**Keywords:** Resilience, Fibromyalgia, Validation, 10-item CD-RISC

## Abstract

**Background:**

No resilience scale has been validated in Spanish patients with fibromyalgia. The aim of this study was to evaluate the validity and reliability of the 10-item CD-RISC in a sample of Spanish patients with fibromyalgia.

**Methods:**

Design: Observational prospective multicenter study. Sample: Patients with diagnoses of fibromyalgia recruited from primary care settings (N = 208). Instruments: In addition to sociodemographic data, the following questionnaires were administered: Pain Visual Analogue Scale (PVAS), the 10-item Connor-Davidson Resilience scale (10-item CD-RISC), the Fibromyalgia Impact Questionnaire (FIQ), the Hospital Anxiety and Depression Scale (HADS), the Pain Catastrophizing Scale (PCS), the Chronic Pain Acceptance Questionnaire (CPAQ), and the Mindful Attention Awareness Scale (MAAS).

**Results:**

Regarding construct validity, the factor solution in the Principal Component Analysis (PCA) was considered adequate, so the KMO test had a value of 0.91, and the Barlett’s test of sphericity was significant (*χ*2 = 852.8; gl = 45; p < 0.001). Only one factor showed an eigenvalue greater than 1, and it explained 50.4% of the variance. PCA and Confirmatory Factor Analysis (CFA) results did not show significant differences between groups. The 10-item CD-RISC scale demonstrated good internal consistency (Cronbach’s alpha = 0.88) and test-retest reliability (r = 0.89 for a six-week interval). The 10-item CD-RISC score was significantly correlated with all of the other psychometric instruments in the expected direction, except for the PVAS (−0.115; p = 0.113).

**Conclusions:**

Our study confirms that the Spanish version of the 10-item CD-RISC shows, in patients with fibromyalgia, acceptable psychometric properties, with a high level of reliability and validity.

## Background

Fibromyalgia is a complex chronic disease, very often difficult to diagnose and treat, and characterized by a set of symptoms including chronic musculoskeletal pain, allodynia, hyperalgesia, physical and psychological fatigue, effort intolerance, sleep disturbance, and morning stiffness [1,2]. Because of the associated high consumption of health resources, high work absenteeism, and high load of suffering for patients and their families, fibromyalgia should be considered an important public health issue [3-6].

Evidence suggests that among individuals with chronic pain such as fibromyalgia, arthritis, or osteoarthritis, psychological factors play a key role in the development and maintenance of the disease [7]. Therefore, in recent years, the number of resilience-related research projects in patients with fibromyalgia has increased [8-10]. Resilience, defined as the ability to recover from and adapt positively to stress and adversity [11], is a multidimensional construct determined by genetic, psychological, social, biological, and environmental factors [11-15], and its variability depends on context, time, age, and life circumstances [16]. Positive emotions have been described as a key aspect of resilience, so that resilient individuals use positive emotions to cope with stress and adverse circumstances [17-20].

Emotional negative affect and distress-related disorders, such as depression or anxiety, are more prevalent in patients with fibromyalgia than in patients with rheumatic diseases [10,21,22], and these patients may also suffer from a relative absence of positive emotional resources [8,23,24]. Nevertheless, evidence suggests that resilient individuals have higher levels of pain acceptance [25] and lower levels of catastrophizing [26,27] and emotional distress [28].

Treatment strategies for fibromyalgia that combine physical activity interventions with cognitive-behavioral treatment in order to foster resilience-related factors have been demonstrated to be equally as or more effective than either single treatment alone [29-34]. Therefore, it is necessary to have valid and reliable instruments for identifying those patients most vulnerable to adverse events, so that they can benefit from these interventions [14,35,36].

Different scales for assessing resilience have been validated in studies of chronic pain population. The Ego-Resilience scale (ER-89) [37] was used to assess resilience in a sample of patients suffering low back pain and osteoarthritis [26]. The Resilience Scale [38] has shown good psychometrics properties in assessing resilience in patients with both spinal chronic pain [39] and musculoskeletal pain [40]. In another study [41], the CD-RISC scale [16] has been used to assess the influence of resilience on depressive symptoms in a sample of spinal cord injury patients, showing that resilience plays an important role in the process of coping with a disability and seems to buffer the occurrence of depressive symptoms.

As far as we know, no resilience scale has been validated in Spanish patients with fibromyalgia. The 10-item CD-RISC [14] is a short version of the CD-RISC scale that has proven good psychometric properties in Spanish [42,43] and Chinese [44] populations. The aim of this study was to evaluate the validity and reliability of the 10-item CD-RISC in a Spanish sample of patients with fibromyalgia.

## Methods

### Study design and population

Our data come from the baseline measurements of an observational prospective multicenter study including patients with diagnoses of fibromyalgia, the methods of which have been published elsewhere [45]. In brief, the sample comprised consecutive patients (N = 208) with fibromyalgia recruited from primary care settings by their general practitioners at the city of Zaragoza, Spain, from January to November, 2010. To be included in the study, patients had to fulfill the American College of Rheumatology criteria for primary fibromyalgia [46] according to a diagnosis made by a Spanish National Health Service rheumatologist and sign an informed consent form. The exclusion criteria were medical or psychiatric disorders that would impede the patient from accurately answering the questionnaire, a diagnosis of a concomitant chronic fatigue syndrome, to be involved in any compensation claims, and poor knowledge of the Spanish language. The overall prevalence of fibromyalgia in adults in Spain is estimated at 2.4 and this syndrome affects more women than men in a ratio of 21:1, increasing with age [47]. This sex ratio is similar to the sample.

The study protocol was approved by the Clinical Research Ethics Committee of Aragon (reference number CP08/07/2009). All participants were asked to give their informed consent orally and in writing, after a full explanation of the research project. After consenting to the study, sociodemographic data were collected, and a battery of questionnaires was administered, which they completed during the visit in which they were assessed at the hospital. This battery included a Pain Visual Analogue Scale (PVAS) for pain intensity and the validated Spanish versions of the 10-item Connor Davidson Resilience scale (10-item CD-RISC), the Fibromyalgia Impact Questionnaire (FIQ), the Hospital Anxiety and Depression Scale (HADS), the Pain Catastrophizing Scale (PCS), the Chronic Pain Acceptance Questionnaire (CPAQ), and the Mindful Attention Awareness Scale (MAAS).

For the reliability analysis, both the 10-item CD-RISC and the FIQ were administered six weeks after the baseline measurement.

### Measurement instruments

#### 10-item CD-RISC

The 10-item CD-RISC [14] is a self-administered questionnaire including 10 items designed as a Likert-type additive scale with five response options (0 = never; 4 = almost always), which had a single dimension. The final score on the questionnaire was the sum of the responses obtained on each item (range 0–40), and the highest scores indicated the highest level of resilience. The Spanish validated version was used [42].

#### Fibromyalgia impact questionnaire (FIQ)

The FIQ [48] is a 10-item self-administered questionnaire developed to measure the health status in patients with fibromyalgia. The first item focuses on the patient’s ability to carry out muscular activities. In the next two items, patients are asked to circle the number of days in the past week they felt good and how often they missed work. Finally, the last seven items are concerned with job performance, pain, fatigue, morning tiredness, stiffness, anxiety, and depression, and are measured by a visual analogue scale (VAS). We used the Spanish version, which psychometric characteristics have been demonstrated (Cronbach α = 0.82; correlations coefficient between differences in health status changes and FIQ scores pre-post treatment = 0.72) [49].

#### Pain catastrophizing scale (PCS)

The PCS [50] is a 13-item self-administered questionnaire that comprises three dimensions: (a) rumination, (b) magnification, and (c) helplessness. There is no established “cut-off” point because pain catastrophizing is considered a personality trait distributed in a continuous way in the general population. The Spanish validated version was used; this version have demonstrated good levels of internal consistency (Cronbach α = 0.79) and test-retest reliability (intraclass correlation coefficient =0.84) [45].

#### Hospital anxiety and depression scale (HADS)

The HADS [35] is a 14-item, self-administered scale with anxiety and depression being assessed by 7 items each. Each item is scored from 0 to 3 with several anchors. Some items are assessed positively and others negatively. A score between 0 and 21 points may be obtained in each domain. The score in each domain may be categorized into four severity groups: normal (0–7), mild (8–10), moderate (15–21), and severe (15–21). The Spanish version of HADS used in this study has evidenced good levels of internal consistency (Cronbach’s α of 0.86 for anxiety and 0.86 for depression) [36]. HADS was selected for use in the present study because it is considered one of the best questionnaires for assessing depression and anxiety in patients with pain disorders [36].

#### Pain visual analogue scale (PVAS)

The PVAS was designed to allow a subjective assessment of pain. A VAS is usually a 10-cm horizontal line, with perpendicular lines on the edges, defined as the extreme limits of pain experience. Anchoring points at each edges are characterized by verbal expressions such as “No pain” (accompanied by the number “0”) at one end and “maximum pain ever experienced” (accompanied by the number “100”) at the other end. Previous studies have demonstrated PVAS to have adequate psychometric properties, including test-retest reliability (r = 0.78) and convergent validity with other pain measures such as McGill Pain Questionnaire (r = 0.49-0.65) [51].

#### The chronic pain acceptance questionnaire (CPAQ)

The CPAQ [52] is 20-item self-administered inventory to asses pain acceptance. There are two principle factors measured by this questionnaire: activities engagement and pain willingness. All items are rated on a 0 (never true) to 6 (always true) scale. The maximum possible total score is 120, with a higher score indicating better acceptance. We used the Spanish validated version that shows adequate psychometric properties such as internal consistency (Cronbach’s α: 0.83) and test-retest reliability (intraclass correlation coefficient: 0.83) [53].

#### The mindful attention awareness scale (MAAS)

The MAAS [54] is a 15-item self-administered questionnaire to assess mindfulness. Principal component analysis (PCA) yielded a one-factor solution. The respondents indicate how frequently they have the experience described in each statement using a 6-point Likert scale from 1 (almost always) to 6 (almost never), where high scores reflect more mindfulness. In an attempt to monitor for socially desirable responses, respondents are asked to answer according to what “really reflects” their experience rather than what they think their experience should be. The items are distributed across cognitive, emotional, physical, interpersonal, and general domains. We used the Spanish version that shows adequate psychometric properties including internal consistency (Cronbach’s α = 0.89) [55].

### Statistical analysis

#### Construct validity

PCA was used to examine the number of factors underlying the scale. Bartlett’s test of sphericity and the KMO index were used to assess the suitability of the factor solution. An eigenvalue of 1 was used as a criterion for factor extraction. To determine the optimal number of factors to retain in the PCA, the interpretability of the factor loadings, Kaiser’s criterion (retention of factors with eigenvalues greater than 1.0), and the scree test were utilized. The scree test involves the examination of a plot of the eigenvalues for breaks or discontinuities. The logic behind the scree test is that the break point divides the important factors from the minor factors.

The suitability of a single factor model underlying the 10-item CD-RISC was analyzed by confirmatory factor analysis (CFA) using IBM SPSS Amos 19 software. Although sex differences in resilience have been described [56], given that the male-to-female ratio is so unbalanced in fibromyalgia, analyses by sex were not considered feasible. The goodness of fit of the hypothetical model to the sample data was assessed according to the Hu and Bentler criteria [57]. To test the factor structure of the 10-item CD-RISC, we randomly split the sample into two subsamples and conducted PCA and CFA in these two sub-samples, respectively.

#### Convergent validity

Partial correlation coefficients were used to examine the relationship between the total 10-item CD-RISC score and other theoretically related constructs used as criterion variables, including chronic pain acceptance, catastrophizing, fibromyalgia, mindfulness, and pain.

#### Reliability

The reliability of 10-item CD-RISC was examined in terms of internal consistency and test-retest reliability. Internal consistency was evaluated by Cronbach’s alpha coefficient using the baseline scores of all questionnaire items. Test-retest reliability was evaluated in the 191 patients in whom the 10-item CD-RISC was administered twice: at baseline and six weeks later. Both the Spearman correlation coefficient and the intra-class correlation coefficient (ICC) between the scores at baseline and six weeks later were used to estimate its reproducibility. The hypothesis that the standard psychometric recommendations for Cronbach’s alpha and ICC were greater than or equal to 0.7 was taken as a starting point for both internal consistency and test-retest reliability [58].

#### Responsiveness

As in other studies [59-62], we used responsiveness as the ability of an instrument to detect differences between two points in time (change over time) within groups. Since resilience is a construct highly influenced by stressful life events, and that for people with fibromyalgia probably the most stressful event on a day-to-day basis is the evolution of their disease, we have tested whether changes in the evolution of fibromyalgia were associated to changes in 10-item CD-RISC as an indirect measure of sensitivity to changes in the 10-item CD-RISC. Therefore, responsiveness was evaluated measuring the test-retest mean differences, effect sizes, and standardized response means (SRM) of the scores on the 10-item CD-RISC scale by categories of change (tertile) on the FIQ scores. We calculated the effect size as follows: ES = (M1 − M0)/SDbas. M0 denotes the mean score of the baseline assessment, M1 the mean score of the follow-up assessment at time 1, and SDbas is the standard deviation of the baseline assessment. SRM was defined as: SRM = (M1 − M0)/SDMxMo, where the numerator is the same as in case of the ES, and the denominator is the standard deviation of the difference in score at baseline and after time 1. Scores of effect size ≥ |0.8| were considered as large effect, scores from ≥ |0.5| to < |0.8| as medium, scores from ≥ |0.2| to < |0.5| as small and scores ≥ |0.1| to |0.2| as trivial [63]. The same cut-off levels were considered for evaluating the SMR, but after transformation in an equivalent of Cohen’s d as follows [63]: d = d’/(√1 − r).

Except for CFA, analyses were performed with IBM SPSS Statistics 19 software.

## Results

Sample characteristics are described in Table 1. The final study included 208 patients, 199 women (95.7%), and 9 men (4.3%), aged 31–66 (mean 52.37, SD: 8.35 years). Each of the subjects described themselves as being of European ethnic origin. The ratio of men to women in the sample was approximately 1:20, similar to the prevalence of FM syndrome sex ratio in population samples. On average, the patients who participated in the study had suffered from FM for 17.58 years (range 1–45; SD: 10.08 years). There were no significant differences in 10-item CD-RISC scores by sex. None of the participants scored 0 on the scale (floor effect), and 1.93% of subjects obtained the maximum score (ceiling effect).

**Table 1:** Sociodemographic characteristics of the study sample (n = 208)

**Mean age (years, SD)**	**52.37 (8.35)**
Age Range	
30-39	21 (10.1%)
40-49	53 (25.5%)
50-59	84 (40.4%)
>60	50 (24.0%)
Sex	
Men	9 (4.3%)
Women	199 (95.7%)
Marital status	
Single	21 (10.1%)
Married	152 (73.1%)
Divorced	28 (13.5%)
Widowed	7 (3.4%)
Education	
No schooling	6 (2.9%)
Primary level	93 (44.7%)
Secondary level	80 (38.5%)
University level	29 (13.9%)
Work status	
Housewife	23 (11.1%)
Unemployed	33 (15.9%)
Employed	50 (24.0%)
Sick leave	30 (14.4%)
Retired	28 (13.5%)
Disable	44 (21.2%)

### Construct validity

**Figure 1:**
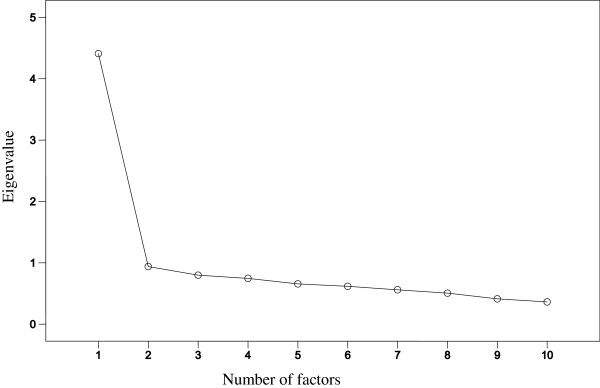
Sedimentation graph of factor components of 10-item CD-RISC.

The factor solution in the PCA was considered adequate, so that the KMO test showed a value of 0.91, and the Barlett’s test of sphericity was significant (*χ*^2^ = 852.8; gl = 45; p < 0.001). Only one factor showed an eigenvalue greater than 1. This factor explained 50.4% of the variance. The saturation of each item in the PCA ranged from 0.87 to 0.89 across the 10 resilience items. The scree plot showed a single suitable factor solution (Figure 1).

### Confirmatory factor analysis

**Figure 2:**
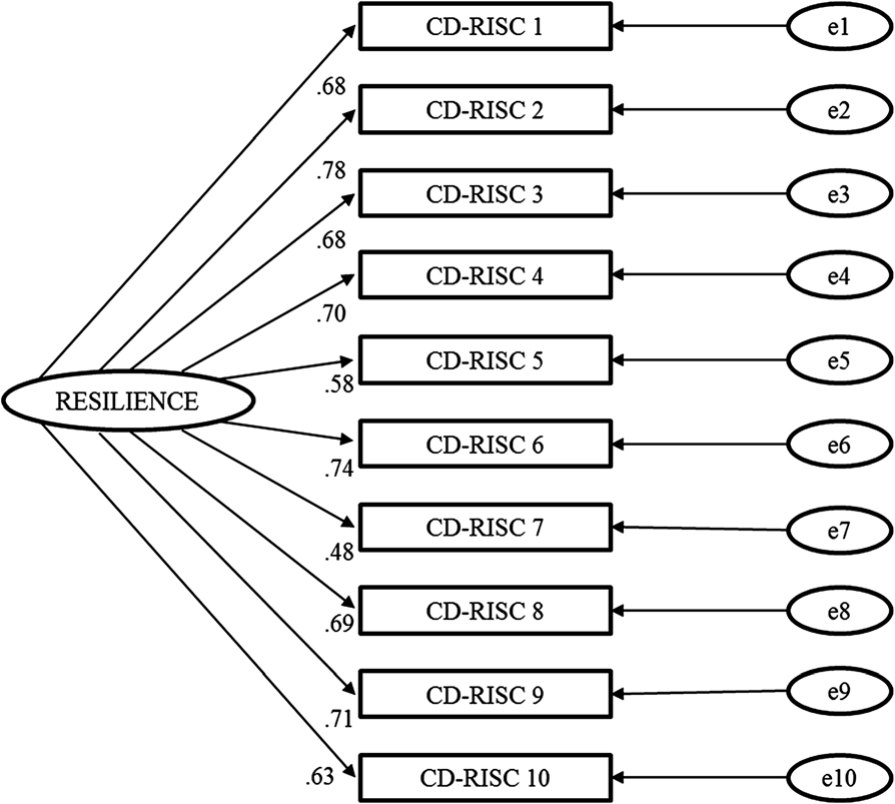
**Factor loading and goodness-of-fit indexes of one-factor model for the 10-items CD-RISC factor structure.** Total sample: n = 208; *χ*^2^ = 68.215; df = 35; p = 0.001; CFI = 0.96; and SRMR = 0.041.

The single factor model proposed for the CFA of the 10-item CD-RISC is shown in Figure 2. The model displayed good fit properties (*χ*^2^ = 68.215, df = 35, p = 0.001; CFI = 0.96, and SRMR = 0.041).

When we randomly split the sample into two groups, the PCA and CFA results did not show significant differences between groups.

### Convergent validity

Partial correlation coefficients were used to assess the relationship between the 10-item CD-RISC and other theoretically related instruments (Table 2). The 10-item CD-RISC score was significantly correlated (negative association with FIQ, PCS, and HADS and positive association with CPAQ and MAS) with all of the other psychometric instruments, except for the VAS (−0.115; p = 0.113).

**Table 2:** Partial correlations among scores of 10-item CD-RISC and measures fibromyalgia impact, pain catastrophizing, anxiety, depression, pain, pain acceptance, and mindfulness (n = 208)

**Variables**	**Correlation**	**P**
FIQ	−0.32	<0.001
PCS	−0.55	< 0.001
HADS-dep	−0.57	<0.001
HADS-ans	−0.51	<0.001
PVAS	−0.12	0.113
CPAQ	0.44	<0.001
MAS	0.39	<0.001

*Abbreviations*: *PVAS* Pain Visual Analogue Scale, *10-item CD-RISC* 10-item Connor Davidson Resilience scale, *FIQ* Fibromyalgia Impact Questionnaire, *HADS* Hospital Anxiety and Depression Scale, *PCS* Pain Catastrophizing Scale, *CPAQ* Chronic Pain Acceptance Questionnaire, *MAAS* Mindful Attention Awareness Scale.

### Reliability

The estimates of Cronbach’s α for the items included in the 10-item CD-RISC was 0.88 and did not increase after eliminating any of the items. Item-total correlation coefficients ranged from 0.25 to 0.63 (median = 0.44). The ICC between scores of the 10-item CD-RISC questionnaire administered at baseline and six weeks later was 0.87 (95% CI = 0.83–0.90), the Spearman correlation coefficient was 0.89 (p < 0.001). The paired Student’s t-test did not find significant differences (p = 0.546) between the mean score of 10-item CD-RISC at baseline and after six-week follow-up.

### Responsiveness

The test-retest mean differences, effect sizes, and standardized response means (SRM) by categories of change (tertile) on the FIQ scores, are shown in Table 3. The effect size of changes in 10-item CD-RISC in patients whose scores in FIQ had improved or worsened ranged from 0.2 to 0.45 and was negligible in those patients whose FIQ score was similar at baseline and at the end of the follow-up. The test-retest mean differences in the 10-item CD-RISC scores by category of change in FIQ score were statistically significant.

**Table 3:** Mean change differences in 10-item CD-RISC scores at baseline and after six weeks by categories of change (tertiles) on the FIQ scores during this follow up

**10-item CD-RISC score**	**FIQ scores**		
	**Worse (n = 61)**	**Similar (n = 67)**	**Better (n = 63)**
Baseline mean (SD)	19.89 (9.33)	26.06 (6.81)	28.27 (8.08)*
Follow-up mean (SD)	17.59 (11.65)	26.04 (6.95)	31.21 (11.02)*
Mean difference (SD)	2.29 (5.14)	0.015 (1.16)	2.93 (6.63)*
Effect size	0.25	0.00	0.36
Standardized response mean	0.45	0.02	0.44
Cohen’s d	0.20	0.00	0.33

*p < 0.001.

## Discussion

Our study confirms that the Spanish version of the 10-item CD-RISC shows, in patients with fibromyalgia, acceptable psychometric properties, with a high level of reliability and validity. The characteristic of our study sample represents the general characteristics of patients with fibromyalgia: middle-aged women with several years of duration of the disorder and on invalidity pensions [64].

The 10-item CD-RISC total score in our sample of patients with FM is lower compared with the young adults’ sample studied in the original validation study [42]. These data are consistent with other studies, which indicate that patients with FM show higher levels of psychological distress and lower levels of resilience than the general population and patients with other rheumatic disease [9,65]. A deficit in the functioning of the hypothalamic-pituitary-adrenal axis in patients with FM might be responsible for the lower levels of the resilience in those patients; additionally, this deficit may exacerbate the symptoms as a consequence of the maladaptive response to stress that many of these patients exhibit [66].

### Construct validity and reliability

All of the statistical (PCA and CFA) and graphical analyses (scree plot) confirm that a single factor underlies the resilience construct measured by the 10-item CD-RISC in patients with FM, which is consistent with other studies [43,44], and with the original Spanish validation study of the 10-item CD-RISC [42]. Additionally, the scale demonstrated good internal consistency (Cronbach’s alpha = 0.88) and test-retest reliability (r = 0.89 for a six-week interval), supporting the stability of the scale. The reliability of the scale was similar to that of the 10-item CD-RISC original Spanish version (Cronbach’s α of the original Spanish version = 0.85 and of the scale in FM patients = 0.88), and the weights in factor analysis were within the range of 0.25–0.63 in our study and within the range of 0.48–0.76 in the original.

### Convergent validity

Our data show a negative association between resilience and mood disorder and, conversely, a positive association between resilience and sense of purpose in life, findings in line with Ruiz-Párraga et al. [40] and Catalano et al. [41] that suggest that resilience may buffer against depressive and anxiety symptoms and have a protective effect on physical and psychological wellbeing.

On the other hand, it has been described that catastrophization increases pain severity, disability and emotional distress [67] and plays a more important role in the pain and depression of women with FMS than in women with other chronic pain conditions [68]. Moreover, previous studies have found that resilient individuals with severe chronic pain tend to report better acceptance of pain than non-resilient individuals with equal levels of pain intensity [27,40]. The lack of association between resilience and VAS could be explained because resilience is more associated with the acceptance and modulation of pain than with the severity of pain as such. However, this hypothesis should be confirmed with specifically designed studies.

### Responsiveness

As mentioned above, to examine the responsiveness of measures of a generic construct such as resilience, which is highly influenced by stressful events that an individual has experienced over lifetime, is a very difficult task, especially when changes in the construct are to be produced in only six weeks. The rationale of resiliency model proposed by Richardson GE [69] suggest that the key to protection from biopsychospiritual homeostasis disruption is how the individual negotiates with life events or stressors. In this model, the individual will adjust the level of homeostasis in four ways: resilient reintegration, that reflects the optimal level of adaptation; homeostatic reintegration, the individual returns to the same level of functioning prior to the life event; maladaptive reintegration, the individual moves to a lower level of homeostasis after the life event; and dysfunctional reintegration, the individual respond reflects dysfunctional behaviors and needs psychotherapy. Since there is not a gold standard to evaluate resilience, and following the Resiliency Model, we hypothesize that the resilience construct should be correlated with fibromyalgia severity levels, and therefore individuals with lower mean scores in 10-item CD-RISC at baseline (close to first quartile) will decrease their resilience levels if the severity of the disease increases.

For this reason, we have linked the measurement of changes in resilience to changes in the development of fibromyalgia measured by the FIQ. Our data confirm the negative association between the changes in the levels of resilience with the course of fibromyalgia syndrome measured by FIQ scores and constitute indirect evidence of responsiveness of 10-item CD-RISC.

Nonetheless, these results should be interpreted with caution given the limitations of this study. First, as in any study using self-report measures, the results may have been influenced by participants’ acquiescence and need for social desirability. Second, we did not asses this tool in populations of patients with other types of chronic pain; thus we did not confirm whether the factor structure is or is not specific to fibromyalgia. Third, the overwhelming proportion of women limits the generalizability of the findings to men though some characteristic of the study sample support that represents fairly well the population of patients with fibromyalgia (i.e. the overall prevalence of fibromyalgia in adults in Spain is 2.4%, and the women/men ratio 21:1, figures similar to those our study) [47].

## Conclusions

Considering that treatments that include interventions for promoting resilience might be effective in patients with FM, our study may have importance from the clinical and research point of view, so that it provides a short and clinically practical tool to assess resilience in Spanish patients suffering from fibromyalgia. Our results confirm the adequate psychometric properties of the 10-item CD-RISC and the factor structure stability across different cultures and populations.

## Competing interests

The authors declare that they have no competing interests.

## Authors’ contributions

BNP, VMV, and JGC are the principal researchers and contributed equally to conceiving the study design, conducting the statistical analysis, and editing the manuscript. ETC, MDSP, and MCPY contributed to drafting the manuscript. All authors read and approved the final manuscript. The manuscript has been translated by a native English speaker for ensuring correct grammatical English.
